# Early versus late BCG vaccination in HIV-1-exposed infants in Uganda: study protocol for a randomized controlled trial

**DOI:** 10.1186/s13063-017-1881-z

**Published:** 2017-03-31

**Authors:** Victoria Nankabirwa, James K. Tumwine, Olive Namugga, Thorkild Tylleskär, Grace Ndeezi, Bjarne Robberstad, Mihai G. Netea, Halvor Sommerfelt

**Affiliations:** 1 Department of Epidemiology and Biostatics, School of Public Health, College of Health Sciences, Makerere University, Kampala, Uganda; 2 Centre for Intervention Science in Maternal and Child Health, Centre for International Health, University of Bergen,; 3 Department of Paediatrics and Child Health, School of Medicine, College of Health Sciences, Makerere University, Kampala, Uganda; 4 Department of Internal Medicine and Radboud Center for Infectious Diseases, Radboud University Medical Center, Nijmegen, Netherlands; 5 Global Women and Children’s Health, Norwegian Institute of Public Health, Oslo, Norway

**Keywords:** BCG, Vaccination, Infants HIV-1-exposed, Nonspecific effects, Trial

## Abstract

**Background:**

Bacillus Calmette-Guérin (BCG) vaccination may have nonspecific effects, i.e., effects on childhood morbidity and mortality that go beyond its effect on the risk of childhood tuberculosis (TB). Though the available scientific literature is mostly from observational studies, and is fraught with controversy, BCG vaccination at birth may protect infants in high-mortality populations against serious infections other than TB. Yet, other studies indicate that giving BCG later in infancy may modify immune responses to non-TB antigens and potentially enhance immunity, potentially also against tuberculosis (TB). It is unclear whether BCG vaccination very early in life offers adequate protection against TB and other infections among HIV-1-exposed children because even those who remain uninfected with HIV-1 show signs of impaired immunocompetence early in infancy. This study will compare BCG vaccination at birth with BCG vaccination at 14 weeks of age in HIV-1-exposed infants.

**Methods:**

This is an individually randomized controlled trial in 2200 HIV-1-exposed infants. The intervention is BCG vaccination within 24 h of birth while the comparator is BCG given at 14 weeks of age. The study co-primary outcomes are severe illness in the first 14 weeks of life, and production of tumor necrosis factor, interleukin (IL)-1β, IL-6 and interferon-γ in response to mycobacterial and nonmycobacterial antigens. The study is being conducted in three health centers in Uganda.

**Discussion:**

A well-timed BCG vaccination could have important nonspecific effects in HIV-1-exposed infants. This trial could inform the development of appropriate timing of BCG vaccination for HIV-1-exposed infants.

**Trial registration:**

ClinicalTrials.gov, identifier: NCT02606526. Registered on 12 November 2015.

**Electronic supplementary material:**

The online version of this article (doi:10.1186/s13063-017-1881-z) contains supplementary material, which is available to authorized users.

## Background

### BCG vaccination and nonspecific effects

Several observational studies suggest that routine childhood vaccines may have effects on childhood morbidity and mortality that are separate from their effects on the incidence of the diseases that they target [1–6]. Importantly, the Bacillus Calmette-Guérin (BCG) vaccine may have effects on morbidity and mortality that cannot be attributed to the vaccine’s protective effect against tuberculosis (TB) [7–11]. A case control study among infants in Guinea-Bissau showed that infants who had received the BCG vaccine were at least 60% less likely to have acute lower respiratory tract infections (ALRI) and respiratory syncytial virus infections [12] than those who had not been vaccinated. A small Malawian study indicated that the likelihood of septicemia among infants with a BCG scar was lower than that among infants without a scar [13]. A Senegalese prospective study analyzing data from two birth cohorts (a total of 11,369 children under 2 years of age) found an association between a combination of BCG/DTP administered simultaneously and reduced mortality [1]. A secondary analysis of data from a randomized controlled trial among low-birth-weight preterm infants indicated that there may be an association between early BCG vaccination and lower perinatal mortality, but not lower infant mortality [9]. In a birth cohort study in Uganda, we previously showed that children between 1 month and 5 years of age who had received BCG had a substantially lower mortality than those who had not [14]. In fact, this association was even stronger in the neonates but we decided not to display this potentially important finding because of the possibility that some of the association could have been due to reverse causality, as vaccinators could to some extent have refrained from giving BCG to sick babies. Other observational studies among older children, adolescents and adults have shown mixed results for the effects of the BCG vaccine against atopy [15–18] and helminthic infections [19–21]. Nonspecific effects of vaccines, are now receiving increased attention, all the more so when the incidence of the TB is decreasing worldwide [22]. In fact, the World Health Organization (WHO) recently established a Strategic Advisory Group of Experts working group on NSEs of vaccines [23].

### Methodological challenges in existing studies

The medical literature on the NSEs of vaccines is fraught with controversy [7]. Much of this controversy stems from interpretational challenges of the observational studies carried out in high-mortality areas, and in heavily burdened health care systems [24, 25]. It has been argued that children with a lower morbidity and mortality are more likely to be vaccinated than higher-risk children, resulting in a spurious nonspecific protective effect of vaccines in observational studies [24, 25]. There is also the possibility of survival bias if death (a common study outcome) is associated with missing vaccination cards. This has been observed in some retrospective studies in Africa where child deaths were quickly followed by destruction or loss of vaccination cards [1, 24, 26, 27]. These selection challenges can be alleviated by well-designed and well-executed randomized controlled trials. However, as most childhood vaccines are part of routine national and international child health programmes, it has been considered unethical to randomize children to not receive BCG. For this reason, the medical literature on NSEs has continued to be overshadowed by controversy and continues to be dependent on initial studies carried out several decades ago when morbidity and mortality patterns, as well as the vaccines themselves, were different from what they are now [24, 25]. Two recent systematic reviews concluded that the currently available evidence, which is mainly from observational studies, was of low quality and reiterated the need for randomized controlled trials [28, 29]. To the best of our knowledge, there are currently no other, long-term, ongoing randomized trials with clinical endpoints similar to ours.

### Immunological reprogramming induced by BCG

In order to strengthen the medical literature, one of the most important aims of the study is to identify any immunological reprogramming induced by BCG, which has been recently suggested to be responsible for NSEs [30]. The immunological responses thought to account for the NSEs of BCG may, in broad terms, be considered to involve two processes: trained innate immunity and heterologous immunity. Trained immunity is a novel concept that describes the functional reprogramming of innate immune cells (such as monocytes, macrophages or natural killer (NK) cells) after an infection or vaccination [31]. The molecular mechanism mediating trained immunity is represented by epigenetic modifications due to changes in histone methylation and acetylation, leading to a looser chromatin structure and increased gene transcription [32]. Similar epigenetic reprogramming has been described in monocytes of individuals vaccinated with BCG [33]. In addition to trained immunity, a second important immunological mechanism that is likely to be involved in the NSEs is heterologous immunity: the capacity of memory T-lymphocytes to respond in an interleukin (IL)-12/IL-18-dependent manner with increased production of cytokines upon stimulation with different stimuli [34].

### Timing of BCG vaccination among HIV-1-exposed children

Based on studies showing a nearly 1000-fold increased risk of disseminated BCG infection among HIV-1-infected infants, recent WHO guidelines consider HIV-1 infection a contraindication to BCG vaccination [22, 35, 36]. However, the practical implementation of this guideline has been difficult as HIV-1 diagnosis at birth (when the vaccine is to be given) is not available in many low- and middle-income countries (LMICs) [35, 37]. As a result, all HIV-1-exposed infants, including those yet to be diagnosed as HIV-1 infected, continue to receive BCG at birth in several LMICs [35, 37]. To circumvent this, an alternative approach has been proposed in which the vaccine is delayed until these infants are diagnosed as not having HIV-1 infection [35, 37].

On the one hand, if vaccinating HIV-1-exposed babies with BCG at birth protects them against serious infections other than TB, i.e., through NSEs [7, 8, 11], delaying vaccination could result in increased morbidity and even mortality. In addition, delaying BCG vaccination to a later time when a definitive diagnosis is made could reduce its coverage [37]. The Ugandan Ministry of Health has decided that all infants, including those with HIV-1 infection, but without symptomatic HIV-1 disease, are to receive BCG.

On the other hand, recent studies put into question the age at which the BCG vaccine is best given to HIV-1-exposed and uninfected children [35, 38–41]. It is presently unclear whether BCG vaccination very early in life provides them with adequate protection against TB and other infections. HIV-1 infection during pregnancy may transiently modulate their responses to the BCG vaccine [38]. It appears that they have an impaired response to purified protein derivative (PPD) and heat-killed BCG in comparison to unexposed children [38]. These effects seem to be greatest among younger HIV-1-exposed uninfected children. A recent study in Brazil found the cellular immune response to BCG among younger HIV-1-exposed uninfected children to be impaired in comparison to their unexposed counterparts [35]. BCG-specific T-cell proliferation and interferon (IFN)-γ concentrations were lower in the younger than in the older HIV-1-exposed uninfected infants and than in the unexposed infants. This signified a delay in immune system maturation of HIV-1-exposed infants. Moreover, a Gambian study showed that children vaccinated at a median age of 14 days were less likely to develop a BCG scar if they were HIV-1-exposed uninfected than if they were HIV-1-unexposed and uninfected [39]. A Rwandese study did not replicate this finding but found that HIV-1-exposed uninfected children vaccinated at birth were substantially less likely than HIV-1-unexposed children to react to a tuberculin skin test at 6 months of age [40]. Other studies have linked negative tuberculin skin tests and absence of BCG scars to high morbidity and mortality in children [41]. These BCG-specific immune abnormalities are consistent with a wider range of other anomalies reported among very young HIV-1-exposed uninfected children and are thought to result from in-utero exposure to the HIV-1 infection and/or antiretroviral drugs. These include: a reduced CD4/CD8 ratio, reduced CD4+ and CD8+ naive T-cell percentages, an increased percentage of activated CD8+ T-cells, augmented percentages of CD3(+)/4(−)/8 (−) (DN) and DN/25(−)/44 (+) [42–44], altered cell-mediated immunity and T-cell maturation [42], reduced IL-12 production that persists until 6 months of age that could possibly be immunosuppressive [45], and several cytokine and other immune abnormalities [42, 43, 45–47]. Importantly, many of these anomalies appear to be transient, disappearing as children grow older.

A small trial in South Africa in which newborn HIV-1-unexposed babies were randomized to receive BCG at birth or at 10 weeks of age, showed that delaying the vaccination resulted in increased numbers of BCG-specific CD4 T-cells, most importantly polyfunctional T-cells co-expressing IFN-γ, tumor necrosis factor (TNF)-α and IL-2, at 1 year of age [48]. A recent study among HIV-1-exposed children reported robust T-cellular responses to *Bordetella pertussis* and tetanus toxoid in infants in whom BCG vaccination was delayed to 8 weeks of age [49]. It is unknown, whether these effects of delayed BCG administration translate into enhanced protection against TB or, for that matter, against other serious infections.

The most appropriate timing for BCG vaccination that maximizes both specific and possible NSEs, particularly in HIV-1-exposed children, is thus presently unknown. This uncertainty compels us to study the NSEs of the BCG vaccine with a design that overcomes the methodological challenges of observational studies.

### Purpose and rationale of this study

A rapid increase in the number of HIV-1-positive pregnant women receiving antiretroviral treatment (ART) to prevent mother-to-child transmission (MTCT) of HIV-1 in the last 10 years [50], coupled with stagnating or increasing adult HIV-1 prevalence in LMICs, has resulted in an increase in the number of HIV-1-exposed uninfected children [51–53]. While data on HIV-1-exposed uninfected children is scanty, available evidence suggests that these children have a higher morbidity and mortality in comparison to their unexposed counterparts [54, 55]. In comparison to unexposed children, HIV-1-exposed uninfected children are more likely to contract the common childhood infectious diseases in severe forms [56–58], more likely to be hospitalized and more likely to be resistant to treatment [59–62]. They are also vulnerable to diseases previously reported among immunocompromised children, such as cytomegalovirus colitis [63], hemorrhagic chicken pox, *Pneumocystis jiroveci* pneumonia and others [64].

Bearing the above considerations in mind, we believe that there is clinical equipoise between early and deferred BCG vaccination in HIV-1-exposed, and probably even in HIV-1-unexposed infants. Moreover, the long-term cost-implications and cost-effectiveness of the alternatives have never been investigated.

It is possible that well-timed BCG vaccination could have important NSEs in HIV-1-exposed children. We therefore propose a parallel-group superiority trial that could inform the development of programmatically appropriate timing of BCG vaccination for HIV-1-exposed infants by measuring the safety, potential benefits or disadvantages, costs and cost-effectiveness of early versus late BCG.

## Methods/design

### Hypotheses

1. Compared to deferring BCG vaccination until 14 weeks of age, BCG administered within the first 24 h of birth leads to at least a 30% relative risk reduction of severe illness^1^ among HIV-1-exposed infants
2. Delayed BCG results in at least a 30% higher production of TNF, IL-1β, IL-6, IL-10, IL-17, IL-22 and IFN-γ in response to mycobacterial (from *Mycobacterium tuberculosis* and PPD) and non-mycobacterial (from *Escherichia coli, Candida albicans* and *Staphylococcus aureus*) antigens compared to when BCG is administered at birth in HIV-1-exposed infants

### Primary objectives

To compare

1. The risk of severe illness in the first 14 weeks of life, and
2. The production of TNF, IL-1β, IL-6, IL-10, IL-17, IL-22 and IFN-γ in response to mycobacterial (from *M. tuberculosis* and PPD) and non-mycobacterial (from *E. coli, C. albicans and S. aureus*) antigens at birth (before BCG vaccination) and at 1, 14, 15 and 28 weeks of age among HIV-1-exposed infants administered BCG at birth with those administered a delayed BCG

### Secondary objectives

To compare:

1. The risk of severe illness from 48 h after randomization until 14 weeks of life among HIV-1-exposed infants administered BCG at birth with those receiving delayed BCG
2. HIV-1-exposed infants who received BCG at birth with those administered a delayed BCG with respect to the occurrence of diarrhea, pneumonia and/or severe illness from 14 weeks to 1 year of age
3. HIV-1-exposed infants who received BCG at birth with those administered a delayed BCG with respect to the occurrence of severe illness during infancy
4. Occurrence of diarrhea, pneumonia and/or severe illness during infancy in HIV-1-exposed infants vaccinated with BCG at birth versus those who receive delayed BCG
5. The mortality from birth to 14 weeks of age in HIV-1-exposed infants vaccinated with BCG at birth versus in those who are yet to receive BCG
6. Infant mortality between HIV-1-exposed babies vaccinated with BCG at birth versus in those who receive delayed BCG
7. Through life-cycle modeling, long-term aggregate health benefits and cost-implications of the delivery strategies, and estimate their cost-effectiveness

To increase the diagnostic specificity, we will also try to capture “clinical sepsis^2^,” where an infant with severe illness and a negative blood culture, or from whom a blood culture could not be taken, has two or more of the following findings: total lymphocyte count (TLC) <5000 cells/mm^3^, absolute neutrophil count <1,500 cells/mm^3^, band to total polymorph ratio >0.2 and both of two C-reactive protein (CRP) levels >1 mg/dl in two specimens taken 24 to 48 h apart. Because we are yet to know in what proportion we can manage to collect the required blood specimens, the comparison between the risk of “clinical sepsis” and/or “confirmed sepsis^3^” between the two trial arms will form part of our secondary analyses. Although it is unclear whether BCG scarring is a marker of immune responsiveness in general and it may be that such scarring is unrelated to protection against TB, it is being captured as a marker of “vaccine take.” We will also explore whether children with a scar are less or more prone to severe illness during infancy. The study will also examine the effect of the timing of BCG administration on infant growth.

## Intervention and co-interventions

The study intervention is an intradermal administration to HIV-1-exposed babies of 0.05 ml Tubervac® BCG vaccine from the Serum Institute of India, the vaccine currently provided by the Ugandan vaccination programme. After randomization within 24 h of birth, infants in one trial arm will receive the vaccine immediately (BCG at birth) while infants in the other arm will receive the vaccine at 14 weeks of age (delayed BCG). Our study nurses, already experts in administering all childhood vaccines, including Tubervac®, will ensure that the vaccine is administered as scheduled.

## Key outcome measures

### Primary outcome measures

1. Severe illness in the first 14 weeks of life
2. TNF, IL-1β, IL-6 and IFN-γ in response to mycobacterial (*M. tuberculosis* and PPD) and non-mycobacterial pathogens (*E. coli, C. albicans* and *S. aureus*) at 1, 14, 15 and 28 weeks of age

### Secondary outcome measures

1. Severe illness from 48 h after randomization to 14 weeks of life
2. Severe illness and/or diarrhea and/or pneumonia between 14 and 52 weeks and between 0 and 52 weeks of life
3. Adverse events up to 52 weeks of life
4. Infant death
5. Growth up to 52 weeks of life
6. BCG scar 12 weeks post vaccination

## Study setting

The study is being conducted in Kawaala Health Center III, Kitebi Health Center III in Kampala district and in Mukono Health Center IV, which is located in Mukono district, 27 km east of Kampala. These centers were chosen based on their proximity to laboratories essential for specimen processing and their high number of HIV-1-infected women attending their antenatal and birth clinics. These three government facilities together conduct at least 1200 deliveries per month.

## Selection of participants, inclusion and exclusion criteria

**Fig. 1:**
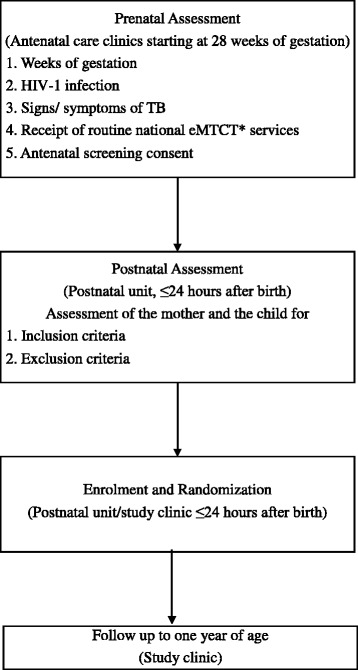
Bacille Calmette-Guérin (BCG) trial participant screening, enrollment and follow-up

HIV-1-infected pregnant women between 28 and 40 weeks of gestation attending antenatal care (ANC) and receiving the national elimination of mother to child (eMTCT) programme’s ART for life (Option B+) strategy are approached by the study team (Fig. 1). ANC clinics in the national programme offer routine opt-out HIV counseling, testing and ART according to the national guidelines and WHO’s Option B+ recommendation. Pregnant HIV-1-infected women attending our three study clinics are informed about the BCG study, its purpose, benefits, risks and their potential eligibility. The study team will encourage the women to deliver at the health units and to follow the national guidelines in order to mitigate MTCT of HIV during pregnancy, childbirth and the breastfeeding period.

For the pregnant women, these guidelines include:

1. Daily, orally administered cotrimoxazole
2. Initiation of triple therapy (tenofovir, lamivudine and efavirenz) starting during pregnancy and continuing for life
3. Appropriately modified obstetric practices
4. Counselling and support for optimal infant feeding

For the HIV-1-exposed infants, these guidelines include:

1. Nevirapine syrup for the first 6 weeks regardless of infant feeding method
2. Daily cotrimoxazole starting at 6 weeks of age

For the HIV-1-infected infants, these guidelines include:

1. Initiation of ART with zidovudine, lamivudine and nevirapine or efavirenz as the preferred regimen.
2. Daily cotrimoxazole starting at 6 weeks

### Inclusion criteria

A baby born at a participating study clinic is included if they:

1. Have a mother with a positive HIV-1 test (ELISA or rapid test)
2. Are receiving periexposure prophylaxis as part of the standard/national Option B+ guidelines in Uganda
3. Have a mother who is of legal age for participation in clinical research studies in Uganda or is an emancipated minor
4. Have a mother/caregiver who resides within the study area, is not intending to move out of the area in the next 4 months and is likely to be traceable for up to 12 months
5. Have a mother/caregiver who gives informed consent to trial participation
6. Have a mother who has received ART for at least 4 weeks

### Exclusion criteria

A newborn child is excluded if they have:

1. Serious congenital malformation(s)
2. Severe illness requiring hospitalization
3. A birth weight <2.0 kg
4. A mother participating in another research study on the day of enrollment or a mother who will participate in another research study within the next 3 months
5. A mother or other household member with symptoms and signs of TB on the day of enrollment
6. A severely ill mother with a condition or conditions requiring hospitalization

## Trial size estimates for the primary hypotheses

### Hypothesis A

The outcome in this hypothesis is severe illness in the first 14 weeks of life. Based on an estimated 10% risk of severe illness in the first 14 weeks of life [65], 90% power and a risk ratio of 0.7, the required sample size is 1914 (Table 1).

**Table 1:** Assumptions used for sample size calculation for the first 14 weeks of life

Assumption	Estimated level
Two-sided significance level	0.05
Ratio of sample size early/late BCG vaccination	1
Percent of vaccinated at 14 weeks of age with severe illness	15
Percent of vaccinated at birth with severe illness	10
Risk ratio:	0.7
Risk difference	5
Total sample size (i.e., in both trial arms)	1914

*BCG* Bacillus Calmette-Guérin

Taking into account 10% attrition and that up to 5% of the infants may have contracted HIV-1 infection when enrolled, a total of 2200 babies are required for this trial, 1100 children in each study arm.

### Hypothesis B

The outcome for this hypothesis is the production of relevant cytokines in response to mycobacterial and non-mycobacterial antigens. Based on previous studies [33, 66] we expect that comparisons involving 50 children in each arm will be sufficient to detect biologically relevant differences.

## Randomization

Infants of HIV-1-infected mothers are randomized within 24 h of birth using pre-prepared randomization lists. Randomization is stratified by clinic using permuted blocks of varying size (4, 6 or 8). Eligible infants are allocated in a 1:1 ratio to one of the two following arms: BCG vaccination within 24 h of birth or BCG vaccination at week 14. All computer-generated randomization lists were prepared for each clinic by an independent scientist, and are kept safely off site. Concealment until the mother-infant pair has been deemed eligible for final inclusion is ensured using an application running on a dedicated Android cell phone at each clinic.

## Blinding/masking

Although scientifically advantageous, giving a placebo injection in the delayed BCG arm may be practically and ethically questionable, and the study is an open-label trial. Rigorously trained [67] research assistants who record outcomes are blinded as much as possible to which trial arm the children belong. Further, the vaccine is administered by routine health workers at the health centers that regularly administer the BCG vaccine as well as other childhood vaccines.

## Implementation plan, staff recruitment and training

### Field organization

Recruitment at each of the three health facilities is done by three full-time research assistants (nurses) and a part-time research assistant. The coordinator (ON) is responsible for the day-to-day running of the study, and will report to the principle investigator (PI; VN) and co-PI (HS) who will ensure the overall scientific integrity of the study. The study will also employ a data manager that will oversee data quality. All study staff have received intensive training on study procedures and refresher training will be conducted during the trial.

#### Screening of pregnant women

All HIV-1-positive pregnant women between 28 and 40 weeks of gestation attending antenatal care at the three clinics are informed about the study. Specifically, they are informed that if they choose to participate in the study, their infants will be randomized to receive the BCG vaccine within 24 h of birth or at 14 weeks of age. Consenting women are offered a CD4 cell count, a full blood count including hemoglobin estimation and a clinical assessment for HIV-1 disease staging and investigations for opportunistic infections, including TB. Pregnant women who are eligible following the screening and who consent to participate are recruited into the study at this first meeting. During recruitment, data on baseline characteristics and other social-demographic variables is collected.

#### Postnatal screening

**Fig. 2:**
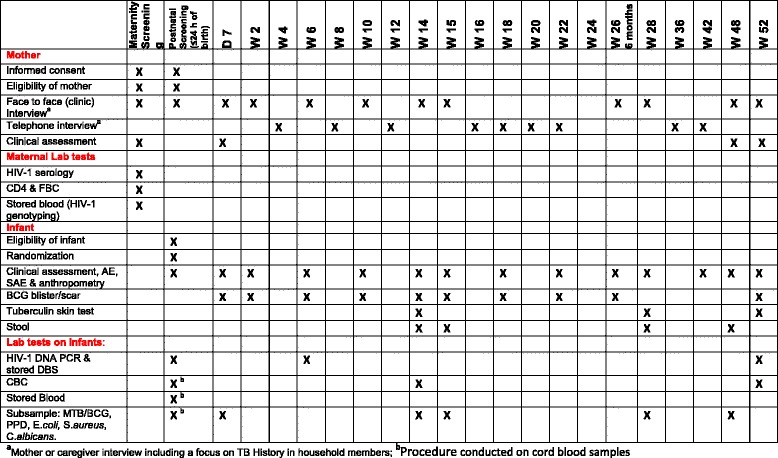
Time schedule for study procedures

Infants are assessed for other inclusion and exclusion criteria, and if eligible, randomized into the study within 24 h of birth. At birth, blood is drawn to perform a deoxyribonucleic acid polymerase chain reaction (DNA-PCR) assay for diagnosing any HIV-1 infection within 6 weeks of birth. The DNA-PCR assays are performed in batches (on samples collected over a 1-month period; Fig. 2) using standard procedures. In addition, cord blood is collected for the child’s full blood count at baseline. A sample of this cord blood is stored for future investigations.

## Collection of data on baseline features and potential confounders

Data on relevant baseline characteristics is collected during pregnancy and at birth. This will include indicators of socio-demographic characteristics (maternal education and age, food insecurity, income, employment, etc.), health (illness history, parity, disclosure of HIV status to a partner, clinical examination, hemoglobin, Body Mass Index (BMI), viral load, CD4 cell count), premature rupture of membranes, postpartum complications, adherence to ART and cotrimoxazole prophylaxis, sex of the child, infant feeding practices, and whether the mother received tetanus toxoid during pregnancy as per national recommendations.

## Follow-up and subject retention

**Fig. 3:**
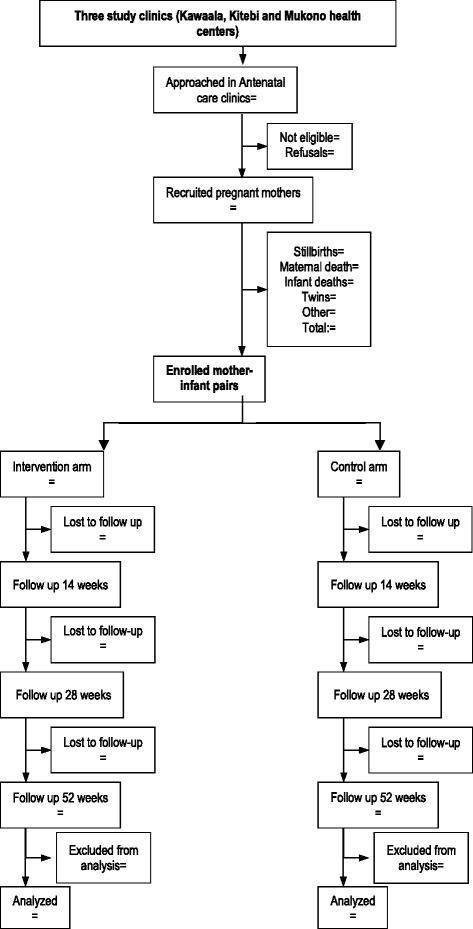
Flow chart of study participants

Follow-up visits at the study clinic are scheduled on day 7 and at weeks 2, 6, 10, 14, 15, 26, 28, 48 and 52 after birth (Figs. 2 and 3). At each of these visits, data is collected on the primary and secondary study outcomes, adverse events (localized abscess formation, suppurative lymphadenitis and disseminated BCG infection), anthropometry and on potential confounding variables (see above). Mothers are given a cell phone to facilitate contact with study staff in case of an illness or other important child events.

Home visits will be made for participants missing clinic visits while those coming to their scheduled clinic visits will have the transport fees reimbursed to reduce losses to follow-up. Data on illness, hospital visits and hospitalization is collected from interviews with the mother, with more detailed information obtained from the hospital records. Illness and hospitalization data is based on recall between a previous visit and a current visit.

Upon enrollment and on subsequent visits, mothers and other caregivers are informed about symptoms and signs of severe illness, and are encouraged to contact the study clinic in case the baby develops any of these symptoms. Each mother is provided with a durable note book, in which she is instructed and trained to note down all relevant events in her child. The front page of the book will have clear reminders that she needs to contact the study team in case the child visits a health clinic, a physician or is admitted to a hospital. A child who comes to a clinic with such symptoms is examined by a study nurse/midwife. If the child does not attend after the mother/caregiver has informed the study team of such signs or for a scheduled visit, the study nurse/midwife will make a visit to the child’s house. If the nurse/midwife classifies the condition as possible severe illness, a study physician will be called to examine the child. If the physician classifies the child as having severe illness, they will collect a blood specimen for blood culture (using BACTEC) and a sepsis screen (CRP, TLC, differential count, and band cell:neutrophil count ratio) as soon as possible. A repeat specimen will be collected again for CRP between 24 and 48 h. The study team will do its utmost to obtain specimens for such a sepsis screen from as many infants with signs of severe illness as possible. However, we have yet to learn what percentage of them in this way can actually be screened for “clinical sepsis.” When 200 enrolled babies have been followed until the age of 6 months, the percentage of infants registered as having had severe illness who are screened for clinical sepsis will be calculated. If this percentage is less than 80%, measures will be taken to increase it above this value, if these measures are unsuccessful, we will need to abandon the attempt to identify clinical sepsis with the sepsis screen. Length and weight measurements are taken twice at each visit, based on the WHO guidelines, and z-scores calculated using WHO child growth standards [68].

## Data collection and management

All trial procedures have been standardized. Training exercises were undertaken and refresher sessions are scheduled during the trial. Using Open Data Kit (ODK, https://opendatakit.org), we will use pre-coded electronic Case Report Forms (eCRFs)/questionnaires with range and consistency checks. Entered data will be checked for consistency and any errors corrected

Source documents and files will not be destroyed without specific written permission from the PI. Only the PI, Co-PI, study coordinator and authorized study personnel will have access to the CRFs and supporting documents that will be kept on password-secured computers to ensure participant confidentiality. To ensure correct operation according to standard operating procedures (SOPs) all system users have been trained in their use and evaluated on a regular basis.

## Clinical laboratory tests

Heel-prick blood samples are obtained from the children for HIV-1 diagnosis. Dried blood spots (DBS) using a commercial PCR kit are used for HIV-1 diagnosis. This testing is supported by standardized protocols that have been successfully validated in Uganda. Flow cytometry is used for CD4 count measurement while standardized automated procedures and techniques are used for hematological and biochemical plasma assays. ELISA and/or rapid tests are used for maternal HIV-1 diagnosis according to the Ugandan national guidelines. At week 14 and week 52, blood will be drawn by venipuncture for full/complete blood counts. TLC, differential count and CRP will be captured as part of the sepsis screen in babies with clinical severe illness.

## Functional immunology assessment

We will investigate both the induction of trained immunity and heterologous immunity. Blood cells will be stimulated for 48 h with a lysate of BCG or *M. tuberculosis*, and IFN-γ production will be measured by ELISA as a measure of classical adaptive immunity. Using the cell culture medium (RPMI) as a control stimulus, trained innate immunity will be assessed by stimulation of blood for 24 h by unrelated nonspecific stimuli: lipopolysaccharide (LPS), heat-killed *E. coli*, *C. albicans* or *S. aureus* at 1, 15 and 28 weeks of age. Production of the monocyte-derived cytokines TNF, IL-1β and IL-6 will be assessed by ELISA. Genome-wide gene transcription will be assessed in the unstimulated and stimulated whole blood by ribonucleic acid (RNA) sequencing. Heterologous immunity will be assessed by measurement of both Th1 (IFN-γ) and Th17 (IL-17, IL-22) cytokines in blood stimulated for 96 h with the nonspecific stimuli described above. The anti-inflammatory cytokine IL-10 will also be assessed at the 48-h stimulation time point. Protein and messenger ribonucleic acid (mRNA) assessment will be our main immunological readouts.

## Quality assurance and quality control

We will use the WHO Good Laboratory Practice Guidelines [69] as our reference when establishing quality control laboratory procedures. HIV-1 serology, CD4 counts, hematology/biochemistry and immunology assays are submitted to a stringent quality assessment programme. On-going training and monitoring will occur during the study.

## Handling losses, withdrawals and protocol deviations

### Protocol deviations

If a protocol violation, such as an inadequate informed consent, inappropriate randomization or concealment or wrongful enrollment of an under-age mother occurs, the PI will inform the Independent Data Monitoring Committee (IDMC), which consists of a pediatrician, a statistician and a pediatrician/immunologist), the Project Management Team (PMT, which consists of some of the authors of this paper) and the local Ethical Committee as soon as possible and no later than five working days after the event. Detailed documentation mentioning the dates and reasons of these protocol deviations will be kept by the PI. If continuing the child in the trial puts the health of the mother or her child at risk, the child will no longer be followed up, but their data will be included in time-to-event analyses up to the time that they were removed from the trial.

## Plan of analysis

The data will be analyzed using Stata version 14 or later (StataCorp LP, TX, USA) and SAS version 9.2 or later. Continuous variables with right-skewed distributions will be log-transformed. Means with standard deviations will be used to summarize symmetrically distributed continuous variables while medians with interquartile ranges will be used for non-normally distributed continuous variables, and percentages for categorical variables. Between-group comparisons for continuous variables that are symmetrically distributed will be made using *t* tests or, if adjustment for other variables are required, linear regression, while Wilcoxon rank sum tests will be used to compare continuous variables where even their log-transformed values remains nonsymmetrically distributed. Group comparisons for categorical variables will use chi-square tests and regression with generalized linear models of the binomial family with a log link (“relative risk regression”) or logistic regression. We will use two-sided statistical tests and 95% confidence intervals for descriptive results, efficacy estimates and safety estimates. All relevant data; from both scheduled and unscheduled visits will be included in the analysis.

### Primary analysis

The main study outcomes are severe illness during the first 14 weeks of life and immunological parameters. Although the sample size estimations are based on specified relative risks, i.e., the occurrence of one or more illness episodes per child, our final analysis will also use incidence density and use Poisson regression or, in case of overdispersion, negative binomial regression analyses, to estimate incidence rate ratios (IRR), enabling us to capture more than one illness event in each child. Further, children who are lost to follow-up will be included in time-to-event analyses, censoring children during periods when data could not be recorded. All randomized children will be included in an *intention-to-treat* analysis.

To take into account multiple study events occurring in the same child, we will in the regression models utilize generalized estimating equations (GEE), as appropriate. The effect of the intervention on the occurrence of severe illness will be estimated using both relative (relative risks (RR), IRR and hazard ratios (HR)) and absolute (risk differences and incidence rate differences (IRD)) measures of effect. In the event that the “relative risk regression” model fails to converge, risk ratios will be obtained from logistic regression [70] or from a modified Poisson regression model with robust variance [71, 72].

Also, autoregressive correlation approaches will be used to account for multiple and possibly correlated observations within the same study participant. These methods take the correlation structures into account and thereby the fact that measurements taken closer in time for an individual are likely to be more correlated than two measurements taken farther apart for that same individual. Efficacy will be calculated as 100 × (1 − RR), 100 × (1 − IRR) or 100 × (1 − HR). The prevalence of BCG scarring will be compared between the two study arms using the abovementioned approaches, as appropriate. Linear regression and or *t* tests will be used to compare the TNF and IFN-γ responses between the trial arms. We will consider adjusting for potential confounders if: (1) there are baseline imbalances between trial arms, (2) the variables for which there are such baseline differences are strongly associated with the study event or (3) they cause a ≥5% difference in the effect measures when they are added to the main model. Full case analyses will be default, but appropriate imputations will be considered for missing data.

In addition to the *intention-to-treat analysis*, where outcomes will be compared according to the random allocation, *instrumental variable analyses* will be conducted in an attempt to estimate biological/causal effects of the actual receipt of the vaccine. In these analyses, random allocation will be the instrument. To enable such analyses, actual receipt as well as the age of BCG vaccination will be captured in all participants. We will also perform *per-protocol* and *as-treated* analyses.

The primary analyses will include all HIV-1-exposed infants, adjusting for any imbalance in HIV-1 status between the two trial arms, as appropriate. However, we will also perform an analysis of the large subgroup of babies who remain uninfected with HIV-1.

### Secondary analysis

1. Risk of severe illness from 48 h after randomization until the first 14 weeks of life, after the 14 weeks of infancy and during the entire infancy period will be analyzed as described above for the primary analysis
2. As a hypothesis-generating effort to understand the latency period after which BCG may induce NSEs, we will calculate the protection against severe illness during the first 14 weeks of life on a sliding scale starting from randomization until seven completed days post randomization
3. Risk of child death in the first 14 weeks as well as in the remaining 38 weeks of infancy: time-to-event analysis will be used to estimate time to severe illness or death. Kaplan-Meier methods and log-rank tests will be used for descriptive analyses. The results from these analyses will be presented as Kaplan-Meier probabilities of the endpoints by a specified time for every 1000 children. Cox proportional hazards regression models will be used to estimate the effect of the intervention on child death. For this outcome, data will be censored when a child is lost to follow-up
4. Risk of clinical sepsis and of “confirmed sepsis” in the first 14 as well as in the remaining 38 weeks of infancy
5. Safety will be analyzed according to type, frequency and severity of adverse events (AEs) that occur in children during the trial
6. Subgroup analysis for potential effect measure modification will be on the strata defined by maternal CD4 counts (<350 or ≥350 cells/μL), low birth weight (<2500 or ≥2500 g) and the babies’ HIV status, sex of the child, in addition to other baseline characteristics which we have reasons to believe may interact with delayed BCG administration. Such subgroup/interaction analyses will be defined based on the available scientific literature before embarking on the analyses, will not be driven by study data, and will be described in a detailed analysis plan
7. Survival analyses using nonparametric and parametric methods to extrapolate over 5 years the incidence of NSEs and death for use in economic evaluation

## Plan of analysis for economic evaluation

**Fig. 4:**
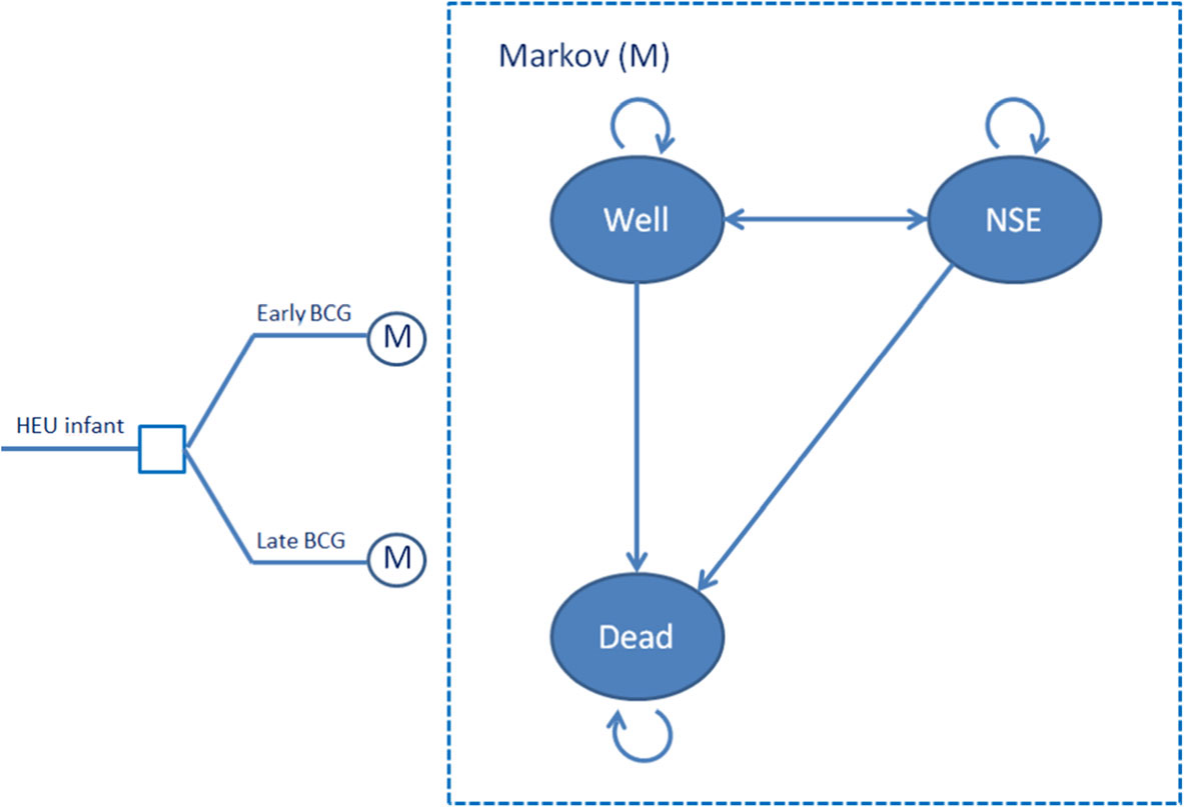
The decision model

A Markov life-cycle decision model will be developed to model long-term aggregate health and cost implications of the delivery strategies, and to compare their cost-effectiveness. The decision model will have two arms, one for early, the other for delayed BCG administration (Fig. 4). A Markov life-cycle will be attached to each delivery arm, in which infants are followed in weekly cycles from 0 to 14 weeks, monthly cycles between 14 and 52 weeks, and thereafter yearly cycles. The model will track one annual Ugandan cohort of HIV-1-exposed infants until they are 5 years old.

The main focus of the model will be severe illness during the first year of life, and will rely on prospectively collected trial data (Table 2). For each cycle (weekly, monthly or annual) children may experience disease, or they may remain healthy. Children aged below 1 year are assumed to be at risk of experiencing a disease other than TB which may persist or from which they recover before the next period. In all health states, individuals may die from that illness or from other causes. At birth, all children are assumed to be alive and well.

**Table 2:** Economic evaluation data requirements and sources

Parameter	Source
Epidemiology	
Age-specific disease incidence	Primary data (for each condition)
Background mortality	Secondary data (Ugandan life table)
Effectiveness	
Disease-specific efficacy	Primary data
Uptake of intervention	Secondary data: effective coverage of vaccination
Aggregate health	
Years of life lost	Secondary data (Ugandan life table)
Disease weights	Secondary data (Burden of Disease Study)
Costs	
Intervention costs	Primary data (prospectively costed)
Treatment costs	Primary data (retrospectively costed)

The model represents a simplification of clinical reality, and a simplifying assumption is that BCG-induced protection against severe illnesses occurs only during the first 5 years of life. This reflects the availability of primary data and follow-up from the controlled trial. Modeling of the health outcomes may be divided into three phases: (a) the observation period, (b) the period of assumed but gradually waning impact of the vaccine (less than 5 years) and (c) the post-vaccine period, where no further vaccination effects are assumed. Extrapolation is required to capture probable health benefits during the second of these phases, but the actual functional shape and other assumptions about continued treatment benefits need to be informed by data through survival analyses, and cannot be decided ex-ante. Transition probabilities are assumed to depend on vaccination strategy, and will be informed by the trial. More specifically, transition probabilities, i.e., probabilities of moving between the health states, will be calculated based on hazard functions from the survival analyses (above). After the extrapolation period, children are not assumed to be protected by BCG, but are assumed to experience background mortality and morbidity for their remaining life time. Long-term health effects will be aggregated using disability-adjusted life years (DALYs) as the instrument, while incremental cost-effectiveness will be expressed in terms of US dollars per DALY averted. Costs will be collected from the perspective of the national health care system, and include those related to providing the BCG vaccine as well as those averted due to the prevention of common illnesses in the short and long term.

## AE reporting/clinical and safety monitoring

An adverse event (AE) is defined as any harmful manifestation occurring in a trial participant, whether this manifestation is related or not to the study BCG vaccine. Potential AEs include localized abscess formation at the injection site, suppurative lymphadenitis and disseminated BCG infection.

## AE monitoring, recording and reporting

Potential AEs will be carefully monitored throughout the trial with specific questionnaires. The mothers will be invited and encouraged to consult the study clinic in case of any disease or symptoms that arise between visits. AEs will be investigated at each follow-up visit. Each AE will be reported spontaneously or in response to general, nondirected discussion with the attending midwife/researcher or physician/researcher. All AEs, regardless of seriousness, severity, or presumed relationship to study therapy, will be recorded using medical terminology in the source document and on the AE page. Whenever possible, diagnoses will be given when signs and symptoms are due to a common aetiology. Investigators will record their opinion concerning the relationship of the AE to BCG vaccination on the AE page. AE reporting

When the investigator, or trained physician, becomes aware that a serious AE (SAE) has occurred, the appropriate reporting form will be completed, and a copy emailed to the local Institutional Review Board.

### Management of SAEs

In case of an SAE, the mother will be encouraged to immediately use the dedicated cell phone to contact the research unit and or to bring the infant immediately to the research unit. Infants will be seen by one of the research midwives/clinicians and appropriate medical or surgical interventions will be provided. All SAEs will be followed up until resolution or until a stable clinical endpoint is reached. Insurance coverage is by BiomedicInsure (http://www.biomedic-insure.com), and if any participant is harmed as a result of the BCG vaccine (within 2 years of the vaccination) they will be compensated

## Monitoring

### Independent Data Monitoring Committee (IDMC)

A group of independent scientists with expertise in pediatrics, immunology, TB and statistics forms an IDMC. After meeting shortly before trial start, it will periodically review and assess available study data for safety, conduct and efficacy. The board will advise the project management on study continuation, modification or termination based on its reviews and pre-established stopping rules.

Interim analysis: an interim analysis for safety taking into account the DAMOCLES group recommendations [73, 74] will be performed by the IDMC when approximately half of all the expected events have been recorded.

### Auditing

The study will be monitored once a year by scientists not involved in the day-to-day undertaking of the trial.

### Protocol amendments

Important protocol modifications (such as those resulting from changes to eligibility criteria, outcomes and planned analyses) will be communicated to, and discussed among, the PMT members, discussed with the IDMC and, when appropriate, the Ethics Committees. Such modifications will also be reflected in amendments to the description in ClinicalTrials.gov (NCT02606526).

### Dissemination of study findings

Our study findings will be communicated in scientific conferences and published in peer-reviewed scientific journals. We will also prepare contextualized evidence briefs for policy to relevant stakeholders including Ugandan authorities (Ministry of Health, National Drug Authority, Uganda National Academy of Sciences) and development partners supporting the Uganda National Expanded Programme on Immunization (UNEPI), WHO (Vaccines and Biologicals) and UNICEF. We will, also in collaboration with CISMAC, prepare plain language summaries and press releases for consumer groups and mass media, respectively. Finally, we will engage the clinic staff and study participants and their communities to inform them of our findings.

## Ethical considerations

According to WHO guidelines, BCG vaccination is contraindicated in children with known HIV-1 infection [22, 35, 36] because of their increased risk of disseminated BCG disease. But implementation of this guideline in many LMICs, including Uganda, is challenging. According to the expanded programme of immunization, the vaccine is to be given as soon as possible after birth. Diagnosing HIV-1 infection in infants requires a PCR which is not accessible to Ugandan children at birth. Therefore, Ugandan HIV-1-exposed newborns receive BCG at birth. On the one hand, receiving the vaccine at birth could be of potential benefit through morbidity and mortality reduction in the initial weeks of life. On the other hand, recent medical literature indicates that HIV-1-exposed uninfected infants [35, 38–41], and maybe even HIV-1-unexposed infants [48], who receive BCG somewhat later may yield more vigorous immune responses than those who receive it at birth. It is conceivable that such a delay translates into better protection against TB. Delaying vaccination may also carry the advantage of increasing the likelihood that HIV-1-exposed infants may be reliably diagnosed with respect to HIV-1 infection, both for logistic reasons but also because there is a genuine risk of HIV-1 transmission via breast milk during the first months of life [75]. This trial will compare BCG take and risk of severe illness (including death) among HIV-1-exposed children receiving the vaccine shortly after birth to such a risk in infants receiving BCG at 14 weeks of age.

The main ethical concerns specific to study are:

1. Acquisition of TB before BCG has engendered a protective immune response (delayed BCG trial arm)
2. Severe BCG disease among HIV-1-positive infants (early BCG trial arm)
3. Higher incidence of severe illness during the first 14 weeks of life among the children who receive delayed BCG

To address the first concern, the study will exclude children with household members who have signs or symptoms of TB or who have a diagnosis of TB. The risk of TB infection among trial babies will, therefore, be negligible [76]. In addition, the study will continue to actively screen and refer suspected cases to the national TB clinics until the participating infants have received BCG (at 14 weeks of age). The study will support diagnosis and initiation of treatment at the TB clinics, of any of the participants’ household members with signs and symptoms of TB from randomization until 14 weeks of age. Moreover, the study team will collaborate closely with the families to identify, treat and actively follow up the babies should there be a history of exposure to active TB or should they develop any symptoms indicative of TB. Should our study show that delayed vaccination is beneficial in terms of protecting HIV-1-exposed babies against severe illness and in the long run it leads to a policy shift, with HIV-1-exposed children from households without TB being vaccinated late, our findings will inform subsequent programmes and would thereby contribute to improved infant health.

To mitigate the second concern, an HIV-1 diagnosis using DNA PCR is obtained at 6 weeks of age. All children who are allocated to receive BCG at birth and are found to be infected with HIV-1 will be referred to a pediatrician for assessment, while the HIV-1-infected participants allocated to receive BCG later will be referred to government clinics for a decision to vaccinate or not. In a sample of 1100 children, the probability of disseminated BCG is extremely low. This is because in the presence of the current Ugandan peripartum ART prophylaxis the risk of HIV-1 infection assessed at 6 weeks of age is less than 3.5% [77]. Therefore, among the 1100 babies who receive BCG at birth, fewer than 40 children are likely to acquire HIV-1. Among children with immunodeficiency, it is estimated that the risk of disseminated BCG infection following vaccination is less than 1.6 per 1,000,000 vaccinated children [78]. Therefore, the likelihood of any child contracting disseminated BCG in our study is less than 0.015% if the government clinics decide to vaccinate all the HIV-1-infected infants at 14 years of age, and 0.007% if they decide not to vaccinate any of them. So, this risk will be equal to, or lower than, that outside of the study, given that Ugandan HIV-1-exposed infants receive BCG shortly after birth. In the study, we shall reduce this risk further with prompt standard TB prophylaxis using isoniazid as soon as an HIV diagnosis is made.

With regard to the third concern, appropriate diagnostic procedures and treatment will be provided according to national guidelines to all severely ill children.

Ethics permission to conduct the study has been obtained from the School of Medicine, Research and Ethics Committee (Makerere University) as well as from the Regional Committees for Medical and Health Research Ethics in Norway (REK) [79]. The trial has also been approved by the National Council of Science and Technology and the National Drug Authority in Uganda. The study is performed in accordance with International Conference on Harmonization (ICH) guidelines for Good Clinical Practice. Written individual informed consent in local language is obtained from each of the participating mothers by trained study staff. The consent process will explain the nature of the study, the risks and benefits of participating in the study, the intervention and that intervention allocation is by a random process. In situations requiring translation or in cases where the mother is unable to read and write, the consent process will take place in the presence of an independent third person, who will act as a witness and also co-sign the Consent Form. Additional consent is obtained from study participants for the collection and storage of blood specimens for ancillary studies. Confidentiality of information and the right of the participant to withdraw from the study at any time during the study is explained to the mothers. All study staff are trained on participant confidentiality and autonomy.

## Discussion

This trial compares the effect BCG vaccination at birth with BCG vaccination at 14 weeks of age in HIV-1-exposed babies on (1) severe illness in the first 14 weeks of life, (2) TNF, IL-1β, IL-6, IL-17, IL-22 and IFN-γ in response to mycobacterial and non-mycobacterial pathogens and (3) severe illness in 14–52 weeks of life and throughout infancy.

The study circumvents the methodological challenges of earlier observational studies that reported an association between BCG vaccination and NSEs. The exclusion criteria combined with the random allocation and large sample size will substantially reduce the likelihood that children with higher morbidity end up in either group. There will be no survival bias linked to missing vaccination cards as BCG is administered before outcome measurement. This study will be further strengthened by an exploration of immunological mechanisms for the vaccine’s hypothesized NSEs. The study will explore the trained innate immunity and heterologous immunity processes that underlie the immunological responses thought to account for the NSEs of BCG.

This trial, comparing NSEs when the vaccine is given at birth and when it is delayed, could inform the development of programmatically appropriate timing of BCG vaccination for HIV-1-exposed infants. This, in turn could importantly impact morbidity and mortality among these infants, who will constitute an increasing part of sub-Saharan African child cohorts in the years to come.

### Trial status

Recruiting since July 2016.

## Additional file

Additional file 1: SPIRIT 2013 Checklist: recommended items to address in a clinical trial protocol and related documents. (DOC 122 kb)

## Abbreviations

AE: Adverse event
BCG: Bacille Calmette-Guérin
CD 4+: Positive to cluster of differentiation 4
CD 8+: Positive to cluster of differentiation 8
CISMAC: Centre for Intervention Science in Maternal and Child Health
CRF: Case Report Form
DNA: Deoxyribonucleic acid
DPT: Diphtheria Pertussis Tetanus
eCRF: electronic Case Report Form
ELISA: Enzyme-linked immunosorbent assay
eMTCT: Elimination of mother-to-child transmission of HIV-1
GCP: Good Clinical Practice
HIV-1: Human immunodeficiency virus-1
IDMC: Independent Data Monitoring Committee
IFN-γ: Interferon gamma
IL-x: Interleukin (x refers to number)
LMIC: Low- and middle-income country
MTCT: Mother-to-child transmission of HIV-1
NDA: National Drug Authority
NK: Natural killer
PCR: Polymerase chain reaction
PPD: Purified protein derivative
SAE: Severe adverse events
TB: Tuberculosis
TNF-α: Tumor necrosis factor alpha
WHO: World Health Organization

## Footnotes

1 Among children aged below 2 months of age, severe illness (other than TB) will be defined as illness that is associated with any of the following danger signs observed or verified by a study clinician: inability to feed or vomiting of everything, lethargy or unconsciousness, severe lower chest in-drawing, axillary temperature of ≥37.5 °C or <35.5 °C, grunting, cyanosis, convulsions or a history of convulsions, and/or results in hospitalization and/or results in death. Among children aged 2 months of age or older, severe illness (other than TB) will be defined as illness that: is associated with at least one of the following danger signs observed or verified by a study clinician: inability to drink or breastfeed, lethargy or unconsciousness, vomiting of all feeds, convulsions or a history of convulsions, and/or results in hospitalization and/or results in death. Hospitalization and death resulting from violent injury or burns will not contribute to the severe illness definition.

2 In infants from whom a blood culture was not performed or in whom a blood culture was negative, clinical sepsis is signaled by one of our study physicians (VN, ON, JKT or GN) having initiated treatment for sepsis or confirmed that such treatment was appropriate and/or by a positive sepsis screen. A positive sepsis screen is the presence of any two of the following: total leukocyte count <5000/mm^3^; absolute neutrophil count <1500/mm^3^; band cell:neutrophil count ratio >0.2; both of two C-reactive protein serum levels in specimens taken 24 h to 48 h apart >1 mg/dl.

3 Confirmed sepsis is signaled by a blood culture that is positive for bacteria known to cause infant sepsis.
